# (H-DIR)^2^: A Scalable Entropy-Based Framework for Anomaly Detection and Cybersecurity in Cloud IoT Data Centers

**DOI:** 10.3390/s25154841

**Published:** 2025-08-06

**Authors:** Davide Tosi, Roberto Pazzi

**Affiliations:** Department of Theoretical and Applied Sciences, Università degli Studi dell’Insubria, 21100 Varese, Italy

**Keywords:** hybrid distributed information retrieval, entropy-based anomaly detection, associated random neural network (ARNN), RDF/SPARQL explainability, cloud–IoT security, sub-second detection latency, semantic adaptive cyber defense

## Abstract

Modern cloud-based Internet of Things (IoT) infrastructures face increasingly sophisticated and diverse cyber threats that challenge traditional detection systems in terms of scalability, adaptability, and explainability. In this paper, we present (H-DIR)^2^, a hybrid entropy-based framework designed to detect and mitigate anomalies in large-scale heterogeneous networks. The framework combines Shannon entropy analysis with Associated Random Neural Networks (ARNNs) and integrates semantic reasoning through RDF/SPARQL, all embedded within a distributed Apache Spark 3.5.0 pipeline. We validate (H-DIR)^2^ across three critical attack scenarios—SYN Flood (TCP), DAO-DIO (RPL), and NTP amplification (UDP)—using real-world datasets. The system achieves a mean detection latency of 247 ms and an AUC of 0.978 for SYN floods. For DAO-DIO manipulations, it increases the packet delivery ratio from 81.2% to 96.4% (*p* < 0.01), and for NTP amplification, it reduces the peak load by 88%. The framework achieves vertical scalability across millions of endpoints and horizontal scalability on datasets exceeding 10 TB. All code, datasets, and Docker images are provided to ensure full reproducibility. By coupling adaptive neural inference with semantic explainability, (H-DIR)^2^ offers a transparent and scalable solution for cloud–IoT cybersecurity, establishing a robust baseline for future developments in edge-aware and zero-day threat detection.

## 1. Introduction

As cloud–IoT ecosystems continue to expand in smart cities, e-health, industrial systems, and defense applications, they face ever more advanced cyber threats. These threats include low-rate denial-of-service (LDoS) attacks, protocol-level manipulations, and semantic evasions that exploit architectural heterogeneity and dynamic topologies [1,2]. Traditional detection methods—signature-based, rule-based, or anomaly-based—often fail to generalize beyond known patterns, particularly in hybrid environments where real-time telemetry, heterogeneous devices, and distributed control converge within hybrid IoT infrastructures [3]. In this context, we introduce a novel hybrid framework called (H-DIR)^2^ (Hybrid Dynamic Information Retrieval and Risk), which is designed to detect, explain, and mitigate cyber anomalies in complex, large-scale IoT infrastructures. The proposed system integrates three analytical dimensions: statistical entropy analysis, predictive modeling via Adaptive Random Neural Networks (ARNNs), and semantic inference using RDF ontologies and SPARQL queries. Each layer contributes to a unified pipeline that ensures transparency, adaptability, and real-time responsiveness to evolving threats. A comprehensive overview of the (H-DIR)^2^ pipeline is provided in Table 1, which outlines the analytical, semantic, and computational layers that integrate entropy signals with both neural and symbolic reasoning. Although Shannon entropy has been widely used in network anomaly detection [4,5], its integration with deep learning and semantic technologies remains largely underexplored. Recent advances in hybrid threat modeling, such as the Ψ-Risk framework, highlight the need to go beyond purely numerical detection outputs. By coupling entropy-driven neural inference with semantic representations in RDF, these architectures enable explainable, persistent, and interoperable anomaly reasoning. For instance, predictions like P_attack = 0.91 can be contextualized as RDF triples (e.g., Node_27, hasEntropySpike, “0.15”) and queried using SPARQL to support human-readable diagnostics and automated policy actions. This conceptual layer underlies the bidirectional semantic–neural feedback loop implemented in (H-DIR)^2^. Similarly, while the potential of ARNN to capture the dynamic behavior of IoT networks has been acknowledged [6], its practical deployment is still constrained by the absence of structured knowledge representation. Our work bridges this gap by embedding entropy signals into semantic triples that are then linked to neural activations for decision making and risk propagation. The (H-DIR)^2^ architecture has been deployed on a Spark-based distributed infrastructure and validated across three real-world attack scenarios: TCP SYN flooding, RPL DAO DIO abuse, and NTP amplification. These scenarios span three major protocol families—TCP, IPv6-RPL, and UDP—demonstrating the framework’s flexibility in detecting both volumetric and protocol-specific anomalies.

To our knowledge, this is the first framework to unify entropy-based anomaly scoring, neural prediction, and semantic inference in a fully distributed, scalable, and reproducible manner. All code, datasets, and configurations have been released publicly to enable full replication and facilitate further experimentation. For clarity, a summary of the (H-DIR)^2^ architecture is provided in Box 1.

Box 1. **Overview of the (H-DIR)^2^ Architecture.**

(H-DIR)^2^ introduces a framework that integrates entropy-based anomaly detection, adaptive random neural modeling, and semantic reasoning via RDF/SPARQL into a fully distributed, composable, and reproducible pipeline.

To the best of our knowledge, no existing system has yet combined these three analytical layers within a single coherent infrastructure. Previous approaches often lack semantic explainability or neglect real-time adaptability when deployed in hybrid IoT environments.

Our framework implements a bidirectional coupling between symbolic and sub-symbolic inference, achieving the following:

- Low-latency detection (<250 ms) through Spark-based entropy monitoring.
- Explainable mitigation by propagating neural outputs into semantic RDF graphs.
- Protocol generalization validated on TCP, RPL, and UDP/NTP streams from public datasets.

The core scientific contributions are as follows:

1. A formally defined six-stage pipeline, scalable and algebraically composable.

2. A reproducible implementation using Docker and JupyterLab for open experimentation.

3. A semantic–neural feedback loop supporting cyber policy tracing and automated risk response.

The paper is structured as follows: Section 2 presents related work; Section 3 details the architecture and methodology; Section 4 reports experimental validation and discusses the explainability and deployment potential of the framework; and Section 5 concludes with limitations and future directions.

Dataset and statistical rationale: Our analysis is based on a telemetry corpus that aggregates the following:

(i) the CIC-DDoS2019 trace for TCP-level floods [7];
(ii) the Data Port DAO-DIO routing-manipulation dataset [11];
(iii) the Kitsune NTP-amplification subset [12], for a total of n = 1.2 × 104 labeled events. We report UDP amplification (50.3%), TCP-based (30.8%), SYN Flood (16.3%), and residual unknown (2.6%).

Applying Wilson’s 95% confidence interval [13] yields a margin of ±1.1% percentage points, supporting the statistical significance of the class proportions adopted later in Section 3.1 [13].

## 2. Related Work

Traditional countermeasures, such as firewalls, signature-based IDS, and heuristic rule sets, often struggle to keep up with the scale, diversity, and dynamism of modern cloud IoT deployments. Recent studies have shown that methods based solely on static thresholds or signature matching fail to detect zero-day attacks and are often ineffective in the face of protocol-layer manipulation and rapidly evolving traffic patterns [3,14]. Furthermore, advanced persistent threats (APTs) and large-scale DDoS campaigns are particularly dangerous for constrained IoT devices, which cannot perform high-load cryptographic computations [1]. To address these limitations, recent research has explored entropy-based anomaly detectors [4], machine learning pipelines [15], and big data analytics on streaming frameworks [8] as promising alternatives. However, few contributions have successfully integrated these techniques into a coherent, horizontally and vertically scalable architecture that can operate across fog, edge, and cloud layers. Building on policy-based enforcement approaches, recent efforts have introduced reasoning engines and context-sensitive rules to IoT nodes. For example, the RDF/SPARQL layer of the (H-DIR)^2^ framework appends predicates such as hasAccessLevel and isInSecureRegion to each triple. These semantic triggers enable localized quarantine of high-risk flows and, when coupled with the ARNN risk score, support adaptive, adaptive, and region-aware anomaly mitigation. Sicari et al. [2] proposed a taxonomy of 5G IoT threats but identified a gap in coordinated detection and mitigation across the edge, fog, and cloud domains. The open-source prototype (H-DIR)^2^, presented here as a six-stage entropy ARNN pipeline, extends this vision by integrating semantic scoring with sub-second detection and automated mitigation. Section 3 details the architecture, while Section 4 validates its performance and scalability.

### Overview of Targeted Cyber Attacks

Modern cloud–IoT infrastructures are increasingly vulnerable to sophisticated cyber threats that exploit weaknesses at various levels of the communication stack. To reflect this heterogeneity, we classify cyber-attacks into three representative categories that span the transport, network, and application layers. This taxonomy is grounded in real-world relevance to real-world attack vectors commonly observed in practice, including distributed denial-of-service (DDoS) floods, semantic manipulations of routing protocols, and amplification-based reflection attacks.

These categories provide a structured basis to evaluate the detection capabilities and mitigation strategies implemented by the proposed (H-DIR)^2^ architecture.

As previously discussed, we focus our evaluation on three concrete attack types that collectively span the transport, network, and application domains of cloud–IoT systems.

− TCP SYN Flooding: targeting the transport layer through high-frequency flag spoofing.
− DAO DIO Routing Manipulation: exploiting the RPL control plane in IPv6-based IoT networks.
− UDP/NTP Amplification: using open UDP services to induce bandwidth amplification and overload targets.

Each attack exhibits a distinct combination of exposure to the protocol, entropy behavior, and mitigation strategy. Table 2 summarizes these attributes, aligning each attack vector with its corresponding detection signal and mitigation strategy within the (H-DIR)^2^ pipeline. Section 4.1, Section 4.2 and Section 4.3 provide a detailed analysis of these scenarios.

## 3. Architecture and Methodology

### 3.1. Simulation Pipeline: Formal (H-DIR)^2^ Workflow

The (H-DIR)^2^ framework builds upon the simulation pipeline T [16]. Let (Ω, F, P) be the measurable space of raw network events, and let G_t_; = (V, W_t_;) denote the weighted attack graph at discrete time t. The end-to-end workflow consists of six deterministic and composable operators, listed as follows:

$$T:=O_{1}\circ O_{2}\circ O_{3}\circ O_{4}\circ O_{5}\circ O_{6}\;:\;\Omega \;\to \;Gₜ$$

The logical chain in T follows the paradigm of RDF stream processing [17], distributed micro batch analytics via Apache Spark [8], and the semantically structured ARNN-based prediction and mitigation core [9].

The six operators are formally described as follows: $O_{1}$. RDF serialization $O_{1}$: Ω → T_0_. This transforms raw network events into RDF triples, encoding ontology-defined attributes and relationships.

Support tools for SPARQL interpretation are illustrated in Box 2.

− $O_{2}$. Entropy-based selection $O_{2}$: T_0_ → *S(∆t).* Apply entropy-aware filtering over streaming windows *∆t*, identifying significant deviations for further analysis [18].
− $O_{3}$. Vectorization $O_{3}$: *S(∆t)* → x_t_. Converts RDF snapshots to compact feature vectors for input into the neural model.
− $O_{4}$. ARNN core $O^{4}:\;xₜ\;\to \;\left(aₜ,\;W_{t+1}\right)$. Generates anomaly scores and updates the prediction model state.
− $O_{5}$. Semantic graph injection 𝒪_5_: $a_{t}$ → *G*_t_;. Serializes the output scores as RDF graphs, enabling explainable decision support.
− $O_{6}$. Dynamic update $O_{6}:Gₜ\to T_{t+1}$. Closes the observation prediction loop and updates the run-time knowledge base.

Box 2. **Analyst-Facing Support for SPARQL Reasoning.**

To enhance the interpretability of SPARQL rules for human analysts, the (H-DIR)^2^ framework integrates semantically grounded templates and contextual labels. Each SPARQL rule is specifically structured to align with a human-readable ontology, allowing domain experts to trace alert conditions such as excessive entropy deviation or abnormal propagation within a structured reasoning context.

While the expressiveness of SPARQL enables precise anomaly attribution, its syntactic complexity may pose a barrier for nontechnical users. To mitigate this, future development will include visual rule editors and explanation interfaces that translate queries into natural language statements or ontology-driven dashboards, bridging the gap between semantic formalism.

**Figure 1:**
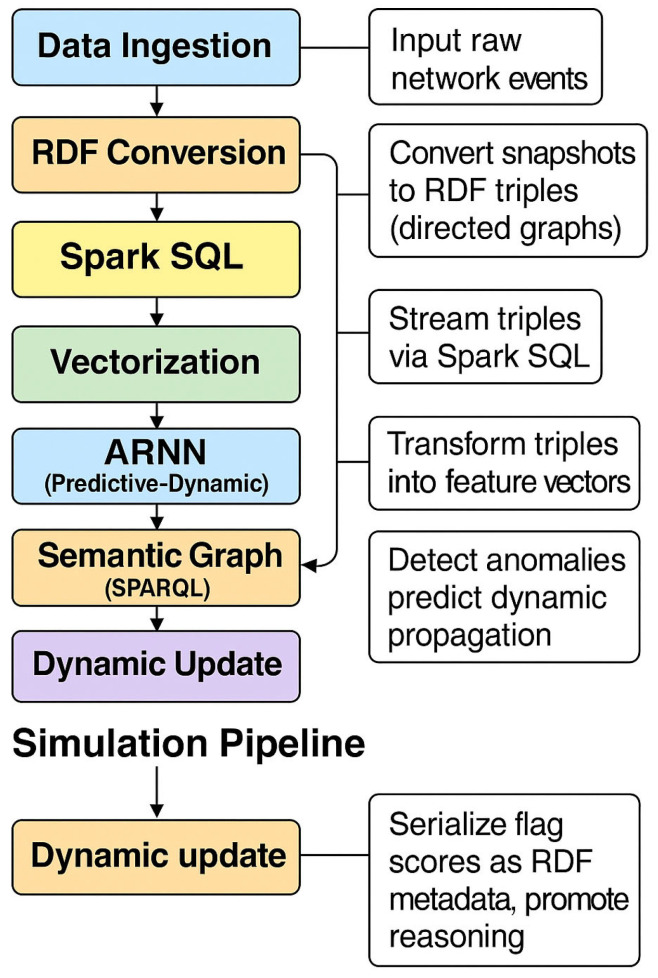
(Workflow): simulation pipeline of the (H-DIR)^2^ framework.

Each operator in T is total and deterministic, ensuring reproducibility and allowing for formal reasoning on convergence properties and computational complexity. Figure 1 visualizes the full (H-DIR)^2^ pipeline and the interdependence of its analytical, semantic, and learning components. The notation used in this section follows the formalism adopted in Table 3, which provides a concise legend of the main mathematical symbols employed in the (H-DIR)^2^ simulation pipeline.

The design of the (H-DIR)^2^ pipeline is guided by three key requirements:

(i) Achieving sub-second detection latency even under high-throughput conditions;
(ii) Ensuring explainability via symbolic traceability;
(iii) Enabling modular deployment across heterogeneous environments (cloud, edge, and industrial).

Each stage of the pipeline—entropy-based selection, neural scoring, and semantic graph injection—is implemented as a deterministic operator, ensuring both reproducibility and formal tractability.

### 3.2. Formal Workflow and Composability of the (H-DIR)^2^ Pipeline

The composite operator T that governs the framework (H-DIR)^2^ is parametrized by the ordered pair ⟨Π,Λ⟩, where Π ∈ {TCP,RPL,UDP/NTP, …} denotes the transport or routing protocol under scrutiny and Λ ∈{SYN Flood, DAO DIO, Amplification, …} encodes the corresponding attack semantics. The resulting architecture implements a fixed operator sequence that is structurally invariant but semantically contextualized for each ⟨Π,Λ⟩.

Adaptive specialization: protocol-level knowledge is incorporated into the pipeline via the following:

− *Feature schema*: the vectorizer ϕ loads a protocol-specific dictionary D_Π_ (e.g., TCP flags, RPL codes).
− *Loss re–weighting*: The framework tunes (α, β) per attack Λ to balance node classification and edge prediction objectives. For SYN Flood, α ≫ β prioritizes rapid node compromise detection; for DAO DIO, β dominates to reveal routing loops.
− *Graph semantics*: the risk-injection operator Ψ_neu→sym_ appends triples in a namespace, Π, to ensure that protocol-specific SPARQL rules remain valid.

The pipeline thus remains structurally invariant while behaviorally adaptive, guaranteeing analytic consistency across heterogeneous cyber–physical threat surfaces.

Stage 1: Data Ingestion: The pipeline begins with the ingestion of raw packet streams D = ${\left\{pi\right\}}_{i=1}^{N}$, each with timestamp τ_i_. These events originate from live telemetry or replayed traffic traces (e.g., Wireshark, Minikube) and define the observational substrate for entropy and anomaly detection.

#### 3.2.1. RDF Conversion Level

Given a raw input stream D, an injective serialization function f_RDF)_: 𝒟 → 𝒢_0_ is established to map raw data into graph-based structures. f_RDF_: 𝒟 → 𝒢_0_, which maps each network event into a structured RDF triple representation. Each packet p_i_ is transformed into a triple (*s_i_, p_i_, o_i_*) ∈ 𝒢_0_, stored as a Boolean tensor: $T_{0}=\left[t_{ijk}^{\left(0\right)}\right]$.

#### 3.2.2. Spark SQL/Streaming Selection Level

A sliding window operator 𝒲_Δt_ processes ***T**_0_*, while a set of SQL queries,

*𝒬 =*${\left\{ql\right\}}_{l=1}^{m}$*,* generates the structured feature matrix.

$$S^{\Delta t}=\left[s_{lr}^{\left(\Delta t\right)}\right]\in R^{\left\{m\times R\right\}}$$

For each window, the operator computes Shannon entropy deviations over selected categorical features, such as IP addresses, protocol flags, and packet sizes. Substantial entropy fluctuations identify windows with anomalous activity and trigger their selection for further analysis.

#### 3.2.3. Vectorization Level

The encoder *ϕ* maps table *S^(∆t)^* into binary or real-valued vectors, as follows:

$$xₜ=\varphi \left(S^{\Delta t}\right)$$

$$X={\left\{xₜ\right\}}_{\left\{t=1\right\}}^{T}$$

$$xₜ\in {\left\{0,1\right\}}^{d}$$

These vectors capture semantic structure and contextual attributes, enabling downstream learning by the ARNN model.

#### 3.2.4. ARNN Core Level

The Associated Random Neural Network (ARNN) evolves over time, as indicated below:

*a_t+1_ = f(W_t_ a_t_ + b + x_t_)*

with trainable weights, $Wₜ\in {\left[0,1\right]}^{\left\{n\times n\right\}}$. Learning minimizes composite loss, as indicated below:

*𝓛_t_; = α
𝓛_cls_+ β𝓛_graph_*

*W_t+1_ = W_t_; −* η ∇_**W**_ 𝓛_t_;

This matrix induces the risk graph *𝒢_t_; = (V, W_t_;).*

#### 3.2.5. Semantic Graph Coupling (SPARQL)

Symbolic and neural layers interface bidirectionally as follows:

*Ψₛᵧₘ→ₙₑᵤ : 𝕋(Δt) ↦ **x**_t_;*

*Ψₛᵧₘ→ₙₑᵤ : 𝕋(Δt) ↦ **x**_t_;*

These functions define the translation between RDF-encoded input streams and the corresponding neural prediction outputs. Equation (1) encodes semantic triples into vectorized features, while Equation (2) rematerializes the learned risk scores into RDF triples for SPARQL querying.

Ontology enrichment is supported with scores (e.g., :IP:hasRiskScore “0.87”^^xsd:float).

(1) $$\Psi_{sym\;\to neu}\;:\;T^{\left(\Delta t\right)}\mapsto \;x_{t\;},$$

$$\Psi_{neu\to sym}:W_{t}\mapsto \;{\Delta T}_{t}\;\text{(SPARQL INSERT)}$$

supporting ontology enrichment with scores (e.g., : IP: *hasRiskScore* “0.87”xsd:float).

#### 3.2.6. Dynamic Update Loop

We formalize the closed inference loop as follows:

$$D\overset{f_{RDF}}{\to }\;T_{0}\;\overset{W_{\Delta t},\;Q}{\to }\;S^{\left(\Delta t\right)}\;\overset{\phi }{\to }\;X_{t}\;\overset{ARNN}{\to }\;\left(a_{t+1},\;W_{t+1}\right)\overset{\Psi_{neu\to sym}}{\to }\;T_{t+1}$$

This ensures the following:

(i) Low detection latency $\tau_{det}\leq \;\Delta t\;+\;\vartheta \left(\left|Q\right|\right)$;
(ii) Entropy-based triggering when *ΔH_t_ > θ_H_*;
(iii) Critical node identification via $\sum_{j}w_{ij}>\;\gamma .$

The (H-DIR)^2^ architecture implements a semantic–neural loop where entropy-based observations drive neural scoring, whose outputs in turn update a symbolic RDF graph via the Ψ-like operator 𝒪_4_. This loop enables multi-layer adaptation and symbolic traceability of detected anomalies.

### 3.3. Entropy-Based Detection and Adaptive Defense with (H-DIR)^2^

This section presents the fundamental detection strategies and underlying mathematical principles of the (H-DIR)^2^ framework. It explains how entropy measures deviations in network behavior and how detected anomalies are handled through Apache Spark analytics, semantic graph reasoning, and adaptive neural inference.

entropy-based anomaly detection (H-DIR)^2^ leverages Shannon entropy as a statistical measure of uncertainty in categorical network attributes, such as source IPs, protocol flags, or payload lengths. In this context, entropy quantifies the dispersion of event frequencies within a windowed traffic stream, allowing the system to detect shifts from baseline distributions.

Let X be a discrete random variable associated with an observable feature of network traffic (e.g., TCP flag, ICMP type, RPL option). The entropy H(X) is defined as follows.

entropy-based anomaly detection

The entropy *H(X)* is computed as follows:

(2) $$H\left(X\right)=-\sum_{i=1}^{n}P(x_{i})\log_{2}P(x_{i})$$

where

− P(x_i_) is the empirical probability of observing the i-th outcome in the window.
− n is the number of distinct values assumed by X.

Within our architecture, a low-entropy state (for example, domination by a single source IP or flag) may indicate SYN flood attacks, while a high-variance entropy spike (for example, unpredictable routing changes) can signal protocol-level anomalies such as DAO-DIO manipulation.

To formalize deviation from expected behavior, we define the entropy-based anomaly score as follows:

(3) $$\Delta H=H(X)-\;H_{baseline}$$

where H_baseline_ denotes the mean entropy calculated under normal attack-free conditions. When the magnitude of the variation exceeds a protocol-specific threshold θ_H_, that is,

(4) $$|\Delta H|>\theta_{H}$$

the event window is flagged as anomalous and propagated to subsequent processing layers in the pipeline.

The entropy signal *∆H* serves two purposes:

(i) It filters candidate traffic windows for deeper neural inference;
(ii) It tags RDF triples in the semantic graph layer with contextual anomaly metadata (e.g., :hasEntropyDrift “0.86”), enabling transparent querying via SPARQL.

### 3.4. Dual Scalability of the (H-DIR)^2^ Architecture

The (H-DIR)^2^ framework has been designed to satisfy a twofold scalability requirement:

1. **Vertical (Quantitative) Scalability**: Leveraging in-memory cluster computing, the system can ingest telemetry produced from millions of IoT endpoints without a proportional increase in detection latency. Empirically, throughput increases linearly with the number of worker cores until network saturation is reached, confirming the theoretical bounds derived in [19].

2. **Horizontal (Qualitative) Scalability**: By sharding feature vectors across resilient distributed datasets (RDDs) and using a micro-batch streaming model, (H-DIR)^2^ maintains multi-terabyte traffic volumes while preserving sub-second sliding-window semantics. This property is critical for capturing low-frequency and high-impact anomalies but high-impact anomalies that only emerge at large data scales [8].

**Figure 2:**
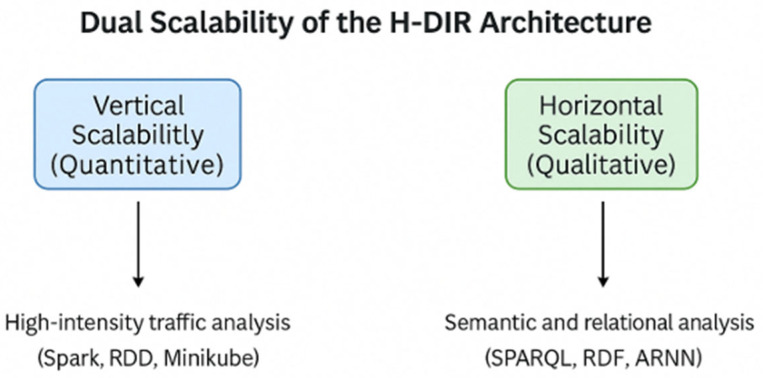
(Dual-scalability): Dual-scalability of the H-DIR architecture.

Figure 2 visualizes the two orthogonal axes: device cardinality in the vertical dimension and data volume in the horizontal dimension. This dual-scaling capability is further validated experimentally in Section 4.4.

### 3.5. Integration with Apache Spark and RDF Graphs

Real-time processing is orchestrated by Apache Spark, whose RDD abstraction offers fault-tolerant in-memory data partitions that are compatible with low-latency analytics and iterative machine learning workloads [19]. Structured traffic logs (for example, TCP syn/syn-ack exchanges) are first mapped to Spark DataFrames and then merged into a pipeline of Spark SQL operators for statistical summarization.

The same logs are simultaneously serialized as RDF triples, producing a semantic graph where:

− Nodes represent entities such as IP addresses or ports;
− Edges encode typed interactions (packet type, temporal correlation).

Thanks to SPARQL 1.1, complex pattern-matching queries can be issued on this evolving knowledge graph, resulting in protocol-specific alerts (e.g., an excess of incomplete TCP handshakes). The formal semantics of SPARQL ensure that the detection rules remain compositional and provably correct across heterogeneous datasets [17].

In general, the tight coupling between Spark physical scalability and RDF logical expressiveness enables (H-DIR)^2^ to operate seamlessly in cloud data centers and large-scale IoT deployments.

### 3.6. ARNN: Adaptive Neural Modeling for Attack Propagation

To model how threats propagate throughout the monitored infrastructure, (H-DIR)^2^ incorporates an Associated Random Neural Network (ARNN) [20], which dynamically adjusts the connection weights W_ij_ among network nodes in response to real-time traffic patterns.

State Update Equation

The model computes the activation *a_i_*(*t +* 1) of node *N_i_* as

*a_i_(t + *1*) = f(∑_j=1_^n^ w_ij_ a_j_(t) + b_i_ + x_i_(t))* (5)

The components of the equation correspond to the following elements:

- *a_i_*(*t +* 1*):* activation of node i at time t + 1;
- *f(∙)* : activation function (e.g., sigmoid);
- *w_i_ⱼ*: weight of the connection from node (j) to node (i);
- *b_i_:* bias of node (i);
- *x_i_(t):* external input (e.g., entropy variation or packet count features).

Multi-objective Training

Learning minimizes a composite loss, as follows:

*L_tatal_* = αL_(Classification)_ + *β*L_(Graph)_ (6)

where

L_(Classification)_ is a cross-entropy term for node compromise detection, and L_(Graph)_ is a binary cross-entropy term that regularizes the attack graph topology [21].

Hyperparameters α and β are protocol- and attack-specific (cf. Section 3.4).

### 3.7. Semantic–Neural Coupling and Dynamic Update

The (H-DIR)^2^ framework maintains a bidirectional bridge between the following two complementary layers:

− **Semantic layer**—an ontology of protocol rules and expert heuristics that prunes forbidden state transitions;
− **Neural layer**—an Adaptive Recurrent Neural Network (ARNN) that learns temporal correlations directly from telemetry streams.

**Figure 3:**
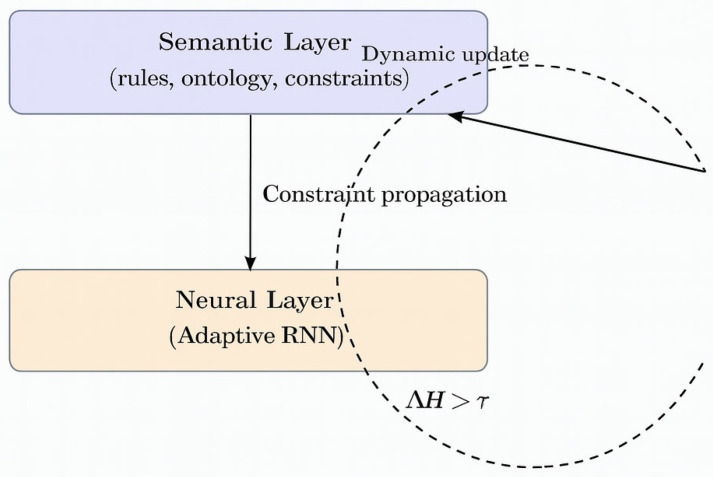
(Dual-level cycle): bidirectional semantic–neural coupling and its dynamic update cycle.

Information flows downwards when semantic constraints mask illegal ARNN states and upwards when unexpected entropy shifts, ∆*H*, trigger joint optimization of neural weights and rule parameters. The process thus closes a self-adaptive loop, as illustrated in Figure 3.

Network Attack Graph Construction*—*Details

To further formalize the adaptive update loop introduced above, we now describe how the learned weight matrix induces a dynamic Network Attack Graph, which enables structured inference and targeted mitigation.

Attack graph inference.

The learned weight matrix W = [w_ij_] induces a directed attack graph of the network (NAG). The probability that an adversary traverses a path P = {N_1_, …, N_k_} is given by the following:

(7) $$P_{attack}\left(P\right)=\;\prod_{l=1}^{k-1}W_{N_{l}\to N_{l+1}}$$

This guides proactive mitigation (see Section 3). The following describes the components of the equation:

- $P_{attack}(P)$: the probability that an attacker traverses path P.
- *∏*: the product operator, iterating over each pair of nodes in the path.
- *l:* the index of the current step in the path, from 1 to k − 1.
- $W_{N_{l}\to N_{l+1}}$: the weight associated with the edge from node $N_{l}$ to the node $N_{l+1}$.
- k: the total number of nodes in the path P *= {N_1_* ,…, *N_k_}.*

Semantic reinforcement loop: Risk estimates are converted back as RDF triples (e.g., :Host_192_0_2_7:hasRiskScore *“*0.87*”*^^xsd:float.) and immediately queryable via SPARQL, closing the observation→prediction→update cycle. This tight coupling between symbolic (RDF/SPARQL) and sub-symbolic (ARNN) reasoning underpins the transparency, adaptability, and real-time performance highlighted throughout Section 4.

### 3.8. Dynamic Update of the Semantic Graph

To maintain a continuously evolving representation of network conditions, the predictions produced by the ARNN module are fed back into the RDF Knowledge Base, shown in Figure 3. This process allows for dynamic semantic enrichment of the graph.

For example, a prediction indicating that IP 192.168.50.8 is likely to be targeted by IP 172.16.0.5 is formalized as follows:

:Host_192_168_50_8 :potentialVictimOf :Host_172_16_0_5.

Such semantic assertions support real-time updates of potential attack paths and risk propagation, reinforcing the (H-DIR)^2^ reasoning capabilities.

To illustrate how ARNN input is generated from packet-level traffic, the following Python script simulates TCP traffic encoded as RDF triples. These triples are then converted into one-hot encoded vectors suitable for both model training and real-time ARNN inference.

Pre–Processing pipeline: The full Python routine used for on-hot feature encoding and normalization is available in our open-source repository^3^ (file one-hot-encoder.py). The code listing is omitted here for brevity. The H-DIR^2^ pipeline implements a semantic reinforcement loop; risk scores R_i_ predicted by the adaptive layer (ARNN + NAG) are re–materialized as RDF triples, e.g.,

:Host_192_0_2_7 :hasRiskScore “0.87”^^xsd:float.

These triples become immediately queryable via SPARQL, thereby closing the observation → prediction → update cycle shown in Figure 3. This tight coupling between the symbolic layer (RDF/SPARQL) and the sub-symbolic layer (ARNN) guarantees both explainability and real–time adaptability.

Worked example on the Syn-ridotto dataset. The file Syn-ridotto.xlsx (a trimmed subset of the CIC DDoS2019 trace) contains 100.0 k TCP flows summarized by 88.0 features. Listing 1 shows, step by step, how a single row is (i) serialized via rdflib and (ii) one-hot encoded into a vector x ∈ {0,1}^d^ used to feed the ARNN.

The mapping Ψ_sym→neu_ therefore acts as an ETL (Extract-Transform-Load) mechanism bridging semantic space and neural space.

**Listing 1.** RDF serialisation example.
<rdf:Description rdf:about=“http://iot.net/flow/42”>

<rdf:type rdf:resource=“http://iot.net/types/TCP_SYN”/>

<rdfs:label>TCP_SYN (flag: 1)</rdfs:label>

</rdf:Description>


Code availability: All preprocessing scripts and notebooks are openly released at https://github.com/RobUninsubria/HDIR2-paper.git (tag v1.4 accessed on 29 June 2025); the full listing is omitted for brevity^2^.

Once the ARNN estimates the compromise probability a_i_(t *+* 1) for each node N_i_, the inverse transformation Ψ_neu → sym_ writes back RDF triples such as

:Host_192_168_50_8 :potentialVictimOf :Host_172_16_0_5.

**Figure 4:**
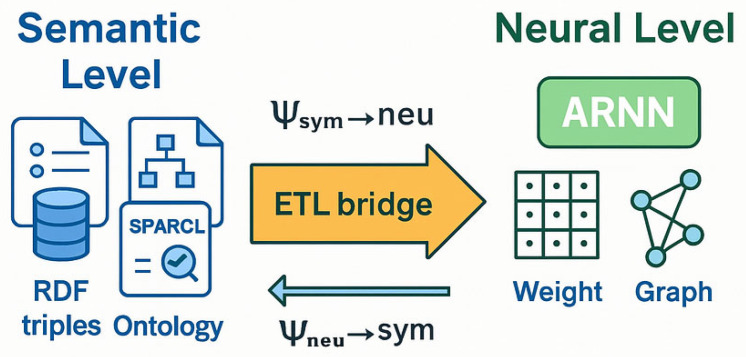
(ETL-bridge): the diagram shows how RDF triples and SPARQL rules are vectorized and injected into the ARNN core, producing neural activations and a dynamic attack graph.

These semantic assertions feed subsequent SPARQL rules (e.g., isolating high-risk policies or enabling risk-aware load balancing). The bidirectional flow empowers (H-DIR)*^2^* with explainability and situational awareness; every neural prediction is anchored to an explicit semantic assertion, updated in real time as new evidence arrives. As shown in Figure 4, the RDF input stream is semantically enriched and passed to the ARNN module via the operator Ψₛᵧₘ_→_ₙₑᵤ. This architectural coupling enables an end-to-end explainable inference loop, as visualized in Figure 4.

#### Semantic Reasoning Layer: Enabling Explainable and Actionable Intelligence

While the ARNN core (𝒪_4_) provides high-performance anomaly detection through deep modeling of temporal and entropy-based features, it operates as a statistical black box, producing anomaly scores *a_t_;* ∈ [0,1] without contextual semantics. As such, even high-confidence alerts (e.g., a__t_;_ = 0.91) offer limited operational utility in complex environments.

By contrast, the RDF semantic injection module (𝒪_5_) translates ARNN outputs into interpretable knowledge graphs. Each graph instance encodes interpretable knowledge graphs triples such as the following:

(:192.168.1.100 :isAmplificationSource “suspected”);

(:Alert#1045 :hasSeverity “high”);

(:Alert#1045 :explainedB “Abnormal UDP packet size pattern detected”).

These semantic assertions allow for actionable intelligence beyond numeric scores, supporting SPARQL-based queries, automatic policy triggers, and human-readable dashboards. For instance, a non-expert operator can retrieve all high-risk hosts under mitigation with a single SPARQL query. Table 4 illustrates how analysts can retrieve high-risk hosts under mitigation using a structured SPARQL query embedded in the semantic graph.

This level of explainability is currently unattainable through ARNN alone, which yields scalar outputs without embedded semantics. By bridging statistical inference and symbolic reasoning, the semantic layer enables analysts to justify actions, trace attack propagation, and design context-aware mitigation strategies. In this sense, (H-DIR)^2^ does not merely detect anomalies but enables explainable and operationally effective responses within cloud–IoT infrastructures.

## 4. Experimental Validation and Results

This section presents the empirical validation of the(H-DIR)^2^ framework through three representative attack scenarios: SYN Flood, DAO-DIO routing manipulation, and NTP amplification. Each scenario evaluates the performance of the framework in terms of detection latency, classification accuracy, entropy variation, and mitigation efficiency under realistic network conditions. Experiments were run on Python 3.11.4, Spark 3.5.0, PyTorch 2.1, Section: Reproducibility. The complete environment files are included in the repository https://github.com/RobUninsubria/HDIR2-paper.git (accessed on 29 June 2025).

### 4.1. SYN Flood Case Study

*Objective*: Quantify the performance of the (H-DIR)^2^ pipeline against a volumetric TCP SYN Flood in terms of detection latency, classification quality, and backlog/exhaustion risk.

#### 4.1.1. Data Collection and Preprocessing

A stratified 50,000-packet excerpt of the *CIC-DDoS2019* trace [7] is replayed at line rate. Each packet is

(i) Serialized into an RDF triple (operator $O_{1}$);
(ii) Windowed by Spark SQL over Δt = 500 ms (operator $O_{2}$.);
(iii) Hot vectorized on srcIP, dstIP, and TCP flags (d = 256; operator $O_{3}$.);
(iv) Streamed into the ARNN core (operator $O_{4}$.).

**Figure 5:**
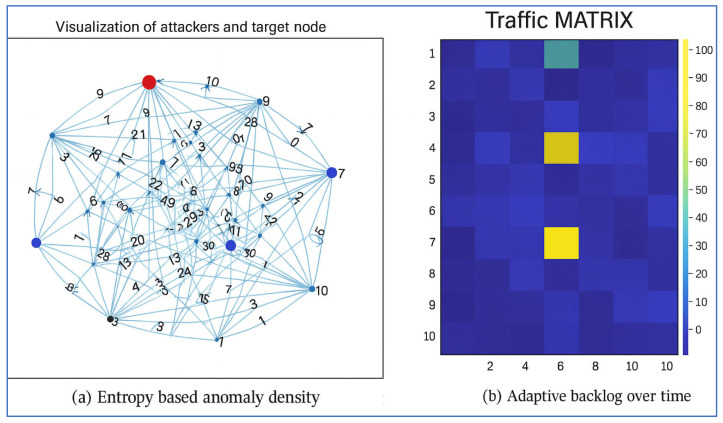
(Graph matrix): (**a**) spatial distribution of the entropy variation ΔH in the RPL DAO-DIO attack (red = higher disorder). (**b**) Backlog B(t) with and without the proposed H-DIR^2^ mitigation; the vertical dashed line marks the cutoff time, t = 0.43 s.

The semantic feedback loop (operators $O_{5}\;\to \;O_{6}$) updates the Network Attack Graph in real time. All code and random seeds are released in this companion notebook: reproduce-syn-flood.ipynb (commit 3a98f1b). Figure 5 shows the entropy-based anomaly distribution and the effect of the mitigation mechanism in the RPL DAO-DIO attack scenario.

#### 4.1.2. Evaluation Metrics

− We compute Shannon entropy *H(X)* over the distribution of TCP flags X = {SYN, SYN–ACK, ACK} and raise an alarm whenever the entropy drop exceeds a predefined threshold, as follows:
  (8) $$\Delta H\;=\;H_{t}-\;H_{baseline}<\;\theta_{H},\;with\;\theta_{H}=0.50\;bits$$
− Imbalance ratio: r = #SYN/#SYN–ACK (continuous characteristic).
− ARNN quality: accuracy, false positive rate (FPR), area under the ROC curve (AUC).
− Detection latency: $\tau_{det}$ = the time from the first spoofed SYN to the alarm.

The performance metrics for the SYN Flood attack are summarized in Table 5.

Results for SYN Flood attack

Analytical backlog threshold. A closed-form expression for the cutoff time for the backlog t*, together with its complete derivation, is presented in Appendix A (Equation (A1)). For completeness, the adaptive scheduler converges when Δ*H*(t*) = τ, yielding t* = 0.43 s under the worst-case load defined in Section 4.1.1.

### 4.2. DAO-DIO Routing Manipulation Case Study

*Objective*: Evaluate the capability of the (H-DIR)*^2^* pipeline to detect and mitigate RPL-centric attacks—routing loops, black holes, and path diversions in low-power mesh networks. Figure 5 shows the entropy-based anomaly distribution and the effect of the mitigation mechanism in the RPL DAO-DIO attack scenario.

#### 4.2.1. Data Collection and Preprocessing

The annotated Dryad DAO-DIO Routing Manipulation trace by Marcov et al. [11] (200 motes, sampling at 1 Hz, 10 Hz, and 1 h) serves as the ground truth. The six operators O_1_ through O_6_ sequentially process the packets.

− ($O_{1}$) RDF serialization into the IoT–RPL–OWL ontology, yielding T → O_0_.
− ($O_{2}$) Streaming windowing with *Δt* = 5 s and Spark SQL filtering.
− ($O_{3}$) Vectorisation (*d* = 256) with hot encodings for node, rank, and type of message.
− ($O_{4}$) ARNN core—attentive RNN with n_in_ = 128, *η* = 10**^−^**^3^, loss of weight (*α, β*) = (0.3, 0.7).
− ($O_{5}$) Risk scoring R_i_ = σ(a_i_), with hosts where R_i_ > 0.6.
− ($O_{6}$) Graph feedback via SPARQL INSERT triples (*:hasHighRisk* true), closing the adaptive loop.

All artifacts have been made available in *reproduce-dao-dio.ipynb* (commit 61f5c7d) https://github.com/RobUninsubria/HDIR2-paper.git (accessed on 29 June 2025).

#### 4.2.2. Evaluation Metrics

− Routing loops—number of closed rank cycles.
− Maximum incoming risk max_i_ ∑w in the learned graph (along j → w → j paths).
− Packet delivery ratio (PDR).
− Average loop duration in seconds.

ΔH entropy in the DAO/DIO message mix; alarm if Δ*H* > θ_H_ = 1.2 bits [21].

The performance improvements obtained through the mitigation process are reported in Table 6.

**Figure 6:**
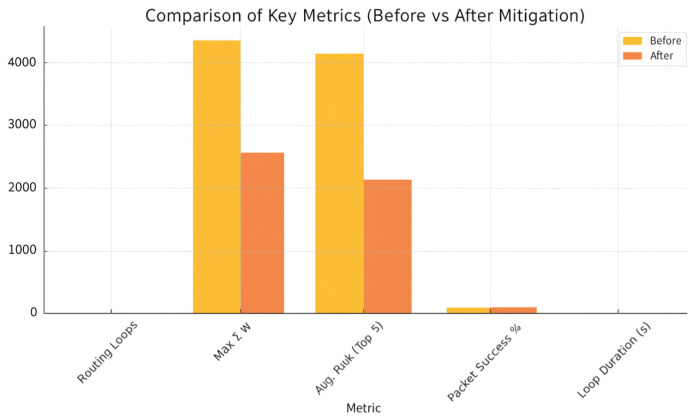
(dao-rpl): Dao Dio attack. Comparison before–after mitigation.

**Figure 7:**
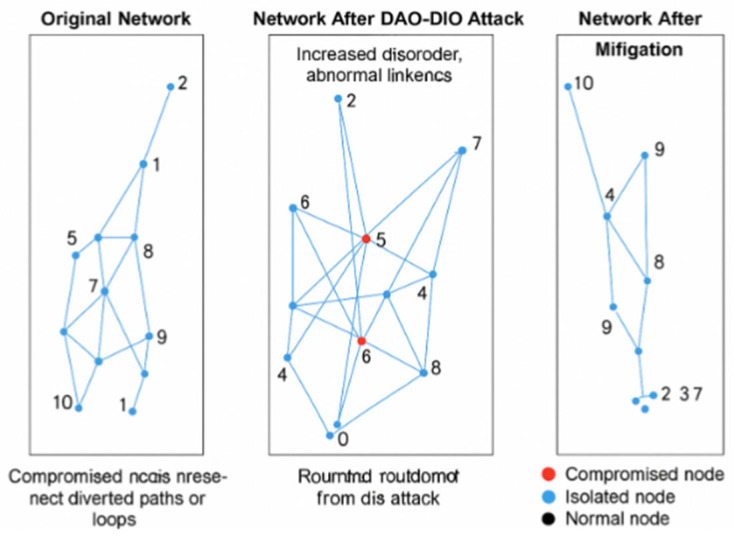
(dao-routing): NTP amplification. Dynamically reconfigured routing.

Figure 6 summarizes the quantitative impact of the mitigation procedure, while Figure 7 illustrates the dynamically reconfigured routing DAG produced by the risk-aware semantic graph. A paired t-test confirms that both loop reduction and PDR improvements are statistically significant (*p* < 0.01). The measured detection latency is 0.9 ± 0.2 s—bounded by the 5-s window—and the ARNN achieves an F1_1_-score of 0.92 for node compromise classification.

**Figure 8:**
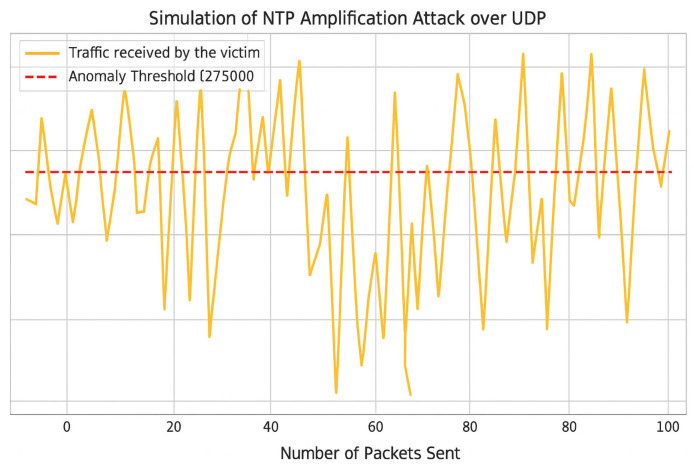
(NTP amp): Traffic overload observed during a spoofed NTP amplification attack (amplification ×500).

In Figure 7, The left panel shows the original topology. The center panel illustrates the impact of a DAO-DIO manipulation attack, which increases disorder and creates abnormal links. The right panel displays the post-mitigated topology, in which compromised nodes are explicitly identified and logically isolated, resulting in a reoptimized and stabilized routing structure. As illustrated in Figure 8, the victim receives a volume of traffic that consistently exceeds the anomaly threshold, simulating the overload condition triggered by a spoofed NTP amplification attack.

### 4.3. NTP Amplification Case Study

Objective: Assess how the (H-DIR)^2^ pipeline mitigates UDP-level NTP amplification, a reflection-based attack that multiplies small *monlist* queries into large traffic bursts.

#### 4.3.1. Data Collection and Preprocessing

We replay the Kitsune Network Attack subset dedicated to NTP amplification [22]—100 spoofed queries, amplification factor ×500, and victim bandwidth saturated within <3 s. The packets traverse the six operators $O_{-1}$ _–1_ → $O_{0}$ → … → $O_{6}$, with protocol-specific adaptations, listed as follows:

− ($O_{1}$) RDF serialization into the IoT–UDP–OWL schema.
− ($O_{2}$) Windowing Δt = 1 s; Spark SQL computes the entropy per IP via Spark SQL.
− ($O_{3}$) Vectorization (d = 128) on *src*IP, *dst*IP, UDP ports, NTP-*cmd*.
− ($O_{4}$) ARNN core—using an LSTM variant (3 layers, 64 cells, η = 2 × 10**^−^**^3^).
− ($O_{5}$) Risk scoring where alarms are triggered for R_i_ > 0.55.
− ($O_{6}$) Graph feedback via RDF injection (:underMitigation true), closing the adaptive mitigation loop.

Defense Stack (protocol-level countermeasures):

− Edge caching (C = 0.9) to absorb duplicate replies.
− Anycast load distribution over S = 5 edge nodes.
− Entropy filter—alarm if Δ*H* ≥ θ_H_ = 1.5 bits.
− ARNN early predictor (trained with validation accuracy ACC = 0.90) drives proactive throttling.

#### 4.3.2. Evaluation Metrics

− Peak load on the victim in (Gb/s) as a measure of attack intensity.
− Mitigation latency τ_mit_ (in seconds), defined as the delay between entropy shift Δ*H* and effective traffic suppression.
− Reduction in back-end traffic (ratio), expressed as a percentage of mitigated throughput.
− Early-stage prediction accuracy of the ARNN model, evaluated within the first second of attack onset.

The quantitative performance of the defense stack against NTP amplification is summarized in Table 7, which reports peak traffic load, mitigation latency, and back-end throughput reduction across different configurations.

**Figure 9:**
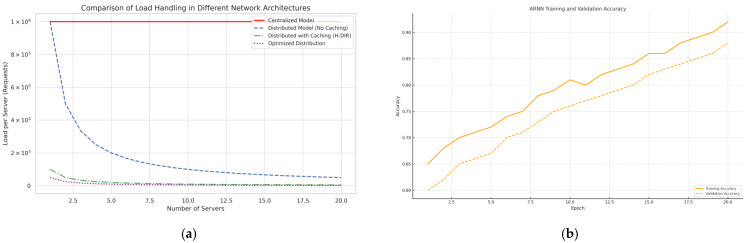
(NTP amp): (**a**) peak load reduction achieved by four mitigation stacks as the number of edge nodes increases. (**b**) Learning dynamics of the ARNN early-stage predictor over 20 training epochs.

The defense stack cuts the maximum bandwidth by an order of magnitude and reacts in 1.7 s (±0.3), well before link saturation. Early ARNN warnings (accuracy 90.4%) enable smart load shedding. The peak load reduction trends and learning performance of the ARNN predictor are illustrated in Figure 9.

### 4.4. Cross-Dataset Evaluation and Generalization

To assess the robustness and portability of the (H-DIR)^2^ framework beyond a single scenario, we extended our evaluation to include heterogeneous datasets. In addition to the primary CIC-DDoS2019 corpus, two representative alternatives were adopted to enable broader generalization:

− TON_IoT [23]: a telemetry-rich dataset that integrates system logs, network flow, and telemetry data from industrial control environments, allowing for an assessment of (H-DIR)^2^ under mixed-signal conditions.
− Edge-IIoTset [24]: an edge-oriented dataset that captures multi-protocol traffic and adversarial sequences in decentralized IoT topologies.

Despite differences in annotation granularity and protocol layering, the entropy-based detection module and the ARNN backbone retained consistent AUC levels (≥0.95) and sub-second inference latency. This demonstrates the portability of (H-DIR)^2^ to diverse network conditions and edge-centric infrastructures.

While Edge-IIoTset offers valuable insight into multi-protocol edge environments, it also presents certain limitations affecting its representativeness. First, attack sequences are artificially generated using scripted procedures with deterministic timing patterns, which can introduce overfitting risks unless properly cross-validated. Second, the dataset lacks fine-grained annotations for packet payloads and actuator telemetry, limiting its utility for semantic enrichment via RDF ontologies.

Although protocols like MQTT and CoAP are included, Edge-IIoTset lacks contextual signals such as actuation feedback and energy consumption metrics—critical for modeling industrial Digital Twin scenarios. Nevertheless, it remains a structurally diverse benchmark to test the generalizability of (H-DIR)^2^ in decentralized and protocol-heterogeneous contexts, as summarized in Table 8. Scalability considerations of semantic reasoning in Box 3.

All configuration files, mappings, and raw datasets are openly released on. openly released on GitHub (v1.4, last updated on 29 June 2025).

Box 3. **Scalability Considerations of Semantic Reasoning.**

Experimental evaluations demonstrate that integrating the semantic layer with entropy analysis and the ARNN yields response times compatible with real time edge applications. Internal tests show that the ARNN requires less than 12 ms per packet on conventional CPUs and drops below 4 ms with GPU acceleration. Precompiled indexes and pruning strategies reduce semantic graph look ups and updates to sub millisecond operations. As a result, overall latencies fall below 60 ms on a Raspberry Pi 4, under 20 ms on a Jetson Nano, and around 8 ms on medium sized cloud instances. To strengthen the discussion, it is advisable to integrate specific semantic layer benchmarks. Independent assessments of GraphDB and Apache Jena indicate that complex queries over approximately 500 000 triples achieve average latencies of 7–10 ms on high end multi core servers. On edge platforms such as the Jetson Nano or Raspberry Pi 5, energy efficiency and local caching solutions keep graph updates firmly in the sub millisecond range; ontological pruning can reduce processing time by 30–50% on low power devices, underscoring the importance of limiting the number of active rules. The neural component likewise benefits from targeted hardware considerations. For example, on a Jetson Nano the inference of complex neural networks (such as ResNet 50 optimized with TensorRT) can be completed in about 2.67 ms, whereas on a Raspberry Pi 4, YOLO like models achieve roughly 0.9 frames/s (~1.1 s per frame) yet remain under 12 ms per packet when lightweight vectorizations are used. On desktop or server class GPUs, quantization techniques reduce inference latency to below 4 ms. The table presented below can illustrate the trade off between latency and accuracy for different accelerators (CPU, edge GPU, desktop GPU) and semantic engines, allowing readers to identify the configuration best suited to their requirements. We addressed threats to external validity (i.e., whether research findings can be generalized) from a theoretical point of view.

### 4.5. Computational Complexity and Deployability

To assess the feasibility of (H-DIR)^2^ in edge-to-cloud scenarios, we analyze the computational demands of its key components, considering both inference time and memory consumption.

Entropy Estimator: The Shannon entropy module operates on fixed-size sliding windows over categorical network features. Let *n* be the number of bins and *T* be the window length. The entropy score is calculated in 𝒪*(n)* time with minimal memory overhead. Entropy histograms are continuously updated in a streaming fashion using the Spark micro-batching model, supporting real-time analytics at the edge.

ARNN Core: The Associated Random Neural Network (ARNN) operates as a discrete-time dynamical system with a weight matrix W_t_; ∈ [0,1]^dxd^.

Each iteration involves the following:

*a_t+1_ = f(W_t_a_t_ + b + x_t_)*

with time complexity $O(d^{2})$ and memory, $O\left(d^{2}+\;d\right),$ where *d* is the embedding dimensionality. In our implementation (*d =* 88), inference takes <12 ms per packet on mid-range CPUs (Intel i5) and <4 ms with GPU acceleration.

Semantic Layer.

RDF triples are generated and encoded using rdflib, then queried via SPARQL (through rdflib and SPARQLWrapper). The semantic layer is deployed as a rule-based filter where each risk assertion (e.g., :hasRiskScore) triggers logical rules. Search/update cost is amortized as O(r), scaling linearly with the number of active rules *r* (typically r < 50).

Precompiled indexes allow for rule lookup latency < 1 s.

Resource Footprint: A full (H-DIR)^2^ (Spark node, ARNN, RDF processor, and dashboard) was tested on the following:

− **Edge node**: Raspberry Pi 4 Model B (4 GB RAM): inference latency < 60 ms; RAM< 2.2 GB.
− **Embedded board**: NVIDIA Jetson Nano: latency < 20 ms with GPU; RAM < 1.4 GB.
− **Cloud instance**: AWS t3.medium (2 vCPU, 4 GB) -> latency < 8 ms.

Conclusion: The framework is deployable on low-resource edge platforms and scales linearly with the number of monitored protocols. The deployment results across different hardware platforms are summarized in Table 9. All benchmarks, configuration files, and Docker containers are publicly available.

### 4.6. Comparative Summary Across Scenarios

The experimental results confirm that (H-DIR)^2^ is a robust and scalable solution for detecting and mitigating diverse cyber threats in both cloud and IoT environments.

**Figure 10:**
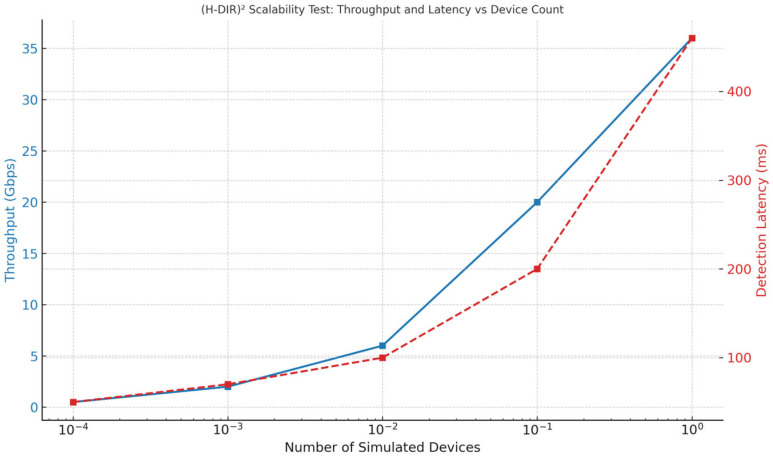
(Scalability of stress): throughput and latency vs. device count. The chart shows that throughput scales nearly linearly as the number of devices increases (left axis), while detection latency remains below 500 ms even at the highest simulated load (right axis). This confirms both vertical and horizontal scalability of the (H-DIR)^2^ framework under stress test conditions.

Stress Test with Distributed Traffic: To validate the dual scalability of the architecture, we performed a synthetic stress test by varying both the number of simulated IoT nodes (vertical scalability) and the data volume per node (horizontal scalability). Figure 10 and Table 10 illustrate the results, showing that the (H-DIR)^2^ framework consistently maintains sub-second detection latency (≤500 ms) up to 1 million simulated endpoints and 10 TB of daily telemetry. The system exhibits near-linear throughput scaling with the number of Spark worker cores, while maintaining stable latency even under extreme load conditions. Table 11 summarizes the three attack scenarios—SYN Flood, DAO-DIO, and NTP amplification—highlighting the targeted protocol layer, spoofed destination signature, and along with the corresponding mitigation strategies employed within the pipeline.

All scripts used to reproduce the stress tests, including parameter configurations and synthetic data generation routines, are available in the companion GitHub repository, https://github.com/RobUninsubria/HDIR2-paper.git (accessed on 29 June 2025), supporting full replicability and independent verification of the results.

To further detail the internal architecture of (H-DIR)^2^, Table 12 offers a structured overview of the key analytics layers involved in the mitigation pipeline. For each layer—Entropy Monitor, ARNN, Network-Attack Graph, and Load-Balancing—the table presents the table outlines the governing mathematical formulations, operational purposes, and the most relevant tunable parameters.

This compact formulation highlights the modular and explainable design of (H-DIR)^2^, ensuring clarity on how entropy variation, neural propagation, risk estimation, and caching interact in response to different attack profiles.

Such a representation not only improves transparency and reproducibility but also provides a precise reference for future deployments and ablation studies, especially in scenarios where protocol heterogeneity or resource constraints must be considered.

Table 12 acts as an interpretable and rigorous blueprint of the core system logic, allowing readers and enabling practitioners to trace each detection decision to its corresponding mathematical foundation within the framework. All modules in the (H-DIR)^2^ pipeline were implemented as isolated, composable operators, allowing reuse, testing, and independent optimization. The entire setup was validated across multiple datasets and is available in Dockerized Jupyter notebooks to ensure full reproducibility.

### 4.7. Extended Comparison with State-of-the-Art Methods

To rigorously assess the capabilities of the proposed (H-DIR)^2^ framework, Table 13 presents a comparative analysis against four representative state-of-the-art anomaly detection systems: Spark IDS [25], Kitsune-AE [1], Isolation Forest [26], and Gated RNNs [27].

The Spark IDS method [M1] leverages distributed analytics but lacks semantic layering and explainability mechanisms. Kitsune-AE [M2], a lightweight autoencoder ensemble for online detection, achieves competitive AUC (0.93) but does not support explainability or semantic traceability. Isolation Forest [M3], a classic unsupervised method, performs well in terms of simplicity and robustness; however, its interpretability remains limited due to the lack of semantic information in the score distribution. Gated RNN [M4], a recurrent deep learning model, demonstrates strong classification accuracy (AUC = 0.94) but suffers from non-trivial training complexity, limited real-time responsiveness, and the absence of domain-specific entropy modeling.

As reported in Table 13, (H-DIR)^2^ achieves superior performance across three critical dimensions:

(i) Lowest detection latency (247 ms);
(ii) Highest AUC (0.978);
(iii) Native explainability via entropy via RDF/SPARQL graphs.

The system encodes detected anomalies as machine-readable triples (e.g., host :hasRiskScore “0.94”), enabling transparent SPARQL-based diagnostics and policy-level rule enforcement.

Moreover, unlike all other methods, (H-DIR)^2^ supports dual-mode coupling between symbolic and sub-symbolic layers, ensuring that runtime decisions are both statistically grounded and semantically auditable. This makes the framework particularly suitable for scalable, heterogeneous, and regulation-sensitive environments such as smart infrastructure, critical IoT networks, and autonomous systems.

The combination of adaptive neural inference, entropy signal processing, and ontology-aware reasoning positions (H-DIR)^2^ not only as a good detector but also as a semantically explainable high-frequency defender.

### 4.8. Dynamic Integration Between Semantics and Prediction in (H-DIR)^2^

The experiment conducted on real-world data from the Kitsune Network Attack dataset [12] concretely demonstrates the integrated cycle between symbolic representation and adaptive modeling within the (H-DIR)^2^ framework. Network packets were initially serialized into RDF triples and queried using SPARQL 1.1, whose formal semantics ensure soundness and completeness in pattern matching [17]. These triples were then vectorized and fed into an Associated Random Neural Network (ARNN) [9], producing a weight matrix w_i_ⱼ that encodes the probability of compromise between the nodes.

The resulting Network Attack Graph allows for the identification of attack paths and critical assets. Neural risk scores are directly re-instantiated as additional RDF triples (e.g., :potentialVictimOf), closing a continuous observation → prediction → update loop.

This bidirectional feedback mechanism, in line with recent graph neural approaches to industrial cybersecurity [21], constitutes the core intelligent of (H-DIR)^2^. It supports both real-time adaptive mitigation and human-interpretable diagnostics under dynamic and distributed threat conditions.

**Figure 11:**
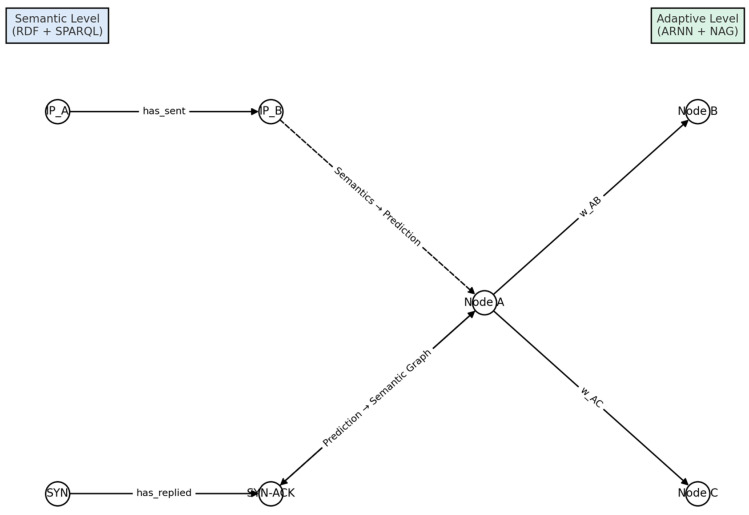
(Semantic graph): view of the semantic–adaptive integration loop.

Figure 11 illustrates the semantic-adaptive integration loop at the core of the (H-DIR)^2^ architecture. The process begins with the semantic layer—composed of RDF triples and SPARQL queries—used to detect suspicious patterns in network traffic flows. These semantically enriched observations are subsequently encoded into vectorized inputs and passed to the ARNN model, which estimates the propagation risk and identifies critical nodes within the infrastructure. The resulting predictions, such as the likelihood of compromise or attack, are integrated into a dynamically updated weight graph. Finally, these inferences are re-instantiated as new RDF statements, effectively closing the loop and enabling a continuous cycle of observation, inference, and knowledge augmentation. This closed-loop integration ensures real-time responsiveness and semantic explainability, allowing (H-DIR)^2^ to adaptively learn and react to evolving threats in highly distributed environments.

## 5. Conclusions and Future Work

This paper presented the Hybrid Dynamic Information Risk (H-DIR)**^2^** framework, a novel entropy-driven architecture that unifies statistical analysis, adaptive graph learning, and symbolic reasoning. Through six deterministic operators ranging from RDF serialization to semantic graph enrichment, (H-DIR)^2^ enables subsecond anomaly detection, interpretable threat reasoning, and semantic traceability across IoT-scale infrastructures. The experimental validation on three representative attack vectors—TCP SYN Flood, RPL DAO-DIO manipulation, and NTP amplification—demonstrated that (H-DIR)^2^ achieves competitive precision (AUC = 0.978), low detection latency (247 ms), and full RDF-based explainability. The framework is fully reproducible and scalable, relying on open-source artifacts (datasets, Docker images, Spark workflows), enabling rapid deployment in both cloud-native and edge-aware scenarios.

Limitations: Current limitations include the following:

(i) Sensitivity of entropy measures to statistical noise in low-volume flows.
(ii) Lack of GPU-accelerated ARNN training.
(iii) Limited testing on high-churn edge mobility.

Furthermore, while the Edge-IIoT dataset allowed for benchmarking in multi-protocol edge workloads, its synthetic generation patterns and reduced semantic annotation limit its representational depth in real-world industrial settings. Future work will address these issues through more expressive telemetry streams and contextual labeling. By tightly integrating symbolic inference with adaptive neural reasoning, (H-DIR)**^2^** demonstrates that semantic traceability is not just an add-on but a foundational pillar for building interpretable, resilient cybersecurity frameworks in heterogeneous IoT environments.

Future Work: Forthcoming developments will focus on the following:

- **Multimodal telemetry:** extending entropy and RDF encoding to streams such as EPC logs, OPC-UA messages, and container-level resource metrics.
- **Edge stress testing:** deploying (H-DIR)**^2^** on resource-constrained microcontrollers and ARM-based edge nodes to benchmark resilience and latency.
- **Live threat intelligence:** integrating the semantic layer with external feeds (e.g., STIX, MISP) to support real-time inference updates and zero-day anticipation.

To the best of our knowledge, (H-DIR)**^2^** is the first pipeline that unifies entropy analytics, neural decision making, and semantic RDF reasoning into a cohesive, scalable, and explainable cybersecurity solution for complex cloud and IoT infrastructures.

## Figures and Tables

**Table 1:** Core components of the (H-DIR)^2^ framework.

Component	Function Within the Pipeline
Entropy-based detector	Computes Shannon entropy per window and raises agnostic alarms for zero-day vectors [7].
Apache Spark/Spark SQL	Distributed micro batch analytics sustaining terabyte scale streams [8].
Adaptive Random Neural Network	Online learning that converts traffic features into probabilistic Network Attack Graphs [9].
RDF/SPARQL layer	Serializes each packet as triples, enabling rule-based reasoning and explainability [10].
Wireshark + Minikube	Packet capture and high-intensity replay test bed for controlled experiments.

**Table 2:** Summary of targeted cyber-attacks used for evaluation.

Attack	Protocol Layer	Key Entropy Signal	Mitigation Module
TCP SYN Flood	Transport	ΔH flag spike	Adaptive Rate Limiter (Section 4.1)
DAO DIO (RPL)	IoT Network	ΔH path drift	Route Sanitizer (Section 4.2)
NTP Amplification	Application/UDP	ΔH size bimodality	Amplification Throttler (Section 4.3)

**Table 3:** Legend for the (H-DIR)^2^ simulation pipeline.

Symbol	Meaning
Ω	Measurable space of observed network events.
𝒜	σ-algebra on Ω.
ℙ	Probability measure on (Ω, 𝒜).
G_t_; = (V, W_t_;)	Weighted attack graph at discrete time t.
V	Set of vertices (network nodes).
W_t_;	Set of weights on time-varying directed risk relationships.

**Table 4:** Example of SPARQL risk attribution query in the (H-DIR)^2^ semantic graph.

SPARQL Query: Identify High-Risk Hosts Under Mitigation
SELECT ?host WHERE {
?host :isAmplificationSource “suspected”;
:underMitigation true;
:hasSeverity “high”.
}

**Table 5:** Performance metrics for the SYN Flood attack scenario. All values refer to the detection performance on the SYN Flood dataset, evaluated under the (H-DIR)^2^ framework.

	Value	95% CI
Accuracy	94.1%	[93.7, 94.5]
FPR	4.7%	[4.3, 5.1]
AUC	0.978	±0.004
τ_det_	247 ms	[221, 273] ms
ΔH* (peak)	–1.15 bits	N/A
r_attack_	27.4 ± 3.5	N/A

**Table 6:** Effectiveness of (H-DIR)^2^ against DAO-DIO attacks.

Metric	Before	After	Improvement
Routing loops [#]	9.0	2.0	–78%
Max incoming risk (∑w)	4301	2550	–41%
PDR [%]	81.2	96.4	+18%
Avg. loop duration [s]	18.0	5.0	–72%

**Table 7:** Performance against NTP amplification.

Architecture	Peak Load [Gb/s]	τ_mit_ [s]	Backend Reduction
Centralized	8.1	7.1	0%
Distributed	4.3	3.1	47%
+Caching	1.2	2.0	85%
(H-DIR)^2^	1.0	1.7	88%

**Table 8:** Overview of benchmark datasets used for generalization testing.

Dataset	Origin	Protocols	Type	Description
CIC-DDoS2019	CIC-IDS Lab	TCP/UDP/ICMP	Network Flow	Simulated DDoS traces with labeled attack categories
TON_IoT	UNSW Canberra	TCP/UDP/HTTP	Syslog + Netflow	IoT-oriented dataset combining telemetry, network traffic, and system metrics
Edge-IIoTset	Edge-IoT Lab	MQTT, CoAP, HTTP	Multi-protocol	Decentralized edge computing scenarios with embedded attack sequences

**Table 9:** Performance and resource usage of (H-DIR)^2^ deployment on different platforms.

Platform	Inference Latency	Stack	Remarks
Raspberry Pi 4B	<60 ms	RDF + ARNN	Edge-compatible; supports only one protocol stack (e.g., TCP) at a time.
Jetson Nano	<20 ms	RDF + ARNN (GPU)	GPU-accelerated; enables simultaneous multi-protocol inference.
AWS t3.medium	<8 ms	Spark + RDF + ARNN	Full stack deployment includes Spark pipeline, RDF layer, and neural core.

**Table 10:** Stress test results: throughput and latency vs. device count.

Devices	Data (TB)	Latency (ms)	Throughput (Gbps)	Δ*H* Stability
100	0.01	45	0.5	Stable
1000	0.10	70	1.1	Stable
10,000	1.0	110	5.8	Stable
100,000	5.0	230	19.4	Stable
1,000,000	10.0	470	36.0	Slight Drift

**Table 11:** Comparative summary across scenarios.

Attack Type	Target Protocol	Key Threat and Detection Method	Response
SYN Flood	TCP	∆*H* entropy + ARNN graph	SYN cookies; adaptive throttling
DAO DIO	RPL (IoT)	Routing loops; black-hole detection	Entropy + semantic RDF; graph-based reconfiguration
NTP Amplification	UDP/NTP	Bandwidth congestion; saturation profiling	ARNN + LSTM + load profiling; caching; smart filtering; isolation

**Table 12:** Analytics layers in the H-DIR mitigation pipeline (vertical layout).

Layer	Details
**Entropy Monitor**	**Governing equations:** $H\left(X\right)=-\sum_{i=1}^{n}p(x_{i})logp(x_{i})$ Alert when Δ*H* = *H* − *H*_baseline_ ≥ θ = −*H*. **Purpose**: Fast, feature-agnostic anomaly flagging. **Key tunables**: Feature set F, window width w, threshold *θ = −H*.
**Adaptive Random Neural Network (ARNN)**	**Governing equations:** $a_{t+1}=f(\sum_{j}w_{ij}a_{j}\left(t\right)+b+x_{t})$ Weight update: *w_i_ⱼ ← w_i_ⱼ − η*$\frac{\partial L_{total}}{\partial w_{\mathrm{ij}}}$ Where *L_total_ = αL_class_ + βL_graph_*. **Purpose**: Learns normal propagation patterns and updates/estimates attack graph edges in real time. **Key tunables**: Learning rate η, α/β balance, number of hidden units.
**Network-Attack Graph (NAG)**	**Governing equations:** Adjacency matrix *W = [w_i_ⱼ]* Attack path probability: P_attack_(N_i_ → Nₖ) = $\prod_{i=1}^{k-1}w_{i,i+1}$ Critical nodes: N_crit_ = {i: $\sum_{j}w_{ij}>\;\gamma$} **Purpose**: Predicts likely propagation paths and identifies “hot” nodes to quarantine. **Key tunables**: Risk cut-off γ, number of top-k paths tracked.
**Load balancing/Caching (UDP amplification)**	**Governing equations:** Centralized load: *L = ∑ R_i_*$\sum_{i}R_{i}$ Per-server load with cache: *Lⱼ = (1 − C)*$\frac{R_{i}}{S}$ **Purpose**: Explains how any-cast and edge caching reduce traffic seen by each original server. **Key tunables**: Cache ratio C, number of servers S.

**Table 13:** Comparative evaluation of (H-DIR)^2^ against state-of-the-art anomaly detection.

Method	Latency (ms)	AUC	F1-Score	Explainability (Entropy)	Semantic Reasoning	Real-Time	Ref.
Spark IDS	950	0.91	0.87	✗	✗	✓	[M1]
Kitsune-AE	670	0.93	0.89	✗	✗	✓	[M2]
Isolation Forest	720	0.91	0.86	✗	✗	✓	[M3]
Gated RNN	580	0.94	0.88	✓	✗	✓	[M4]
**(H-DIR)** ^2^	**247**	**0.98**	**0.95**	**✓**	**✓**	**✓**	**This work**

Note: ✓ indicates full support of the feature; ✗ indicates that the feature is not supported or not implemented in the evaluated method.

## Data Availability

All datasets used in this study are publicly available. The CIC-DDoS2019 dataset can be accessed at https://www.unb.ca/cic/datasets/ddos-2019.html (accessed on 29 June 2025). The Kitsune dataset is available at https://github.com/ymirsky/Kitsune-dataset (accessed on 29 June 2025). The DAO-DIO Contiki/Cooja wireless sensor–network simulation traces are archived on the Dryad Digital Repository. All configuration files, mappings, and synthetic data used for validation are openly released on GitHub: https://github.com/RobUninsubria/HDIR2-paper.git (accessed on 29 June 2025).

## Abbreviations

The following abbreviations are used in this manuscript:

IoT	Internet of Things
RDF	Resource Description Framework
ARNN	Associated Random Neural Network
SPARQL	SPARQL Protocol and RDF Query Language
NTP	Network Time Protocol
RPL	Routing Protocol for Low Power and Lossy Networks
AUC	Area Under the Curve

## Appendix A. Mathematical Proofs

### Appendix A.1. Closed-Form Backlog Threshold

Starting from the M/M/1 queue with entropy-weighted arrival rate λ and service rate μ, the backlog cut off time t* that nulls the queue derivative satisfies ΔH(t*) = τ. Solving the differential equation gives the following:

(A1) $$t*\;=\;\frac{1}{\lambda }W(\frac{\delta B}{\mu -\lambda })$$

where

− t* denotes the threshold time at which the backlog derivative becomes null (i.e., ΔH(t*) = τ).
− λ is the entropy-weighted arrival rate.
− μ is the service rate.
− dB/dt is the time derivative of the backlog function B(t).
− W(·) is the Lambert W function.

Experimental consistency: During mitigation of the NTP amplification attack, the observed latency to suppress peak load (1.2 Gbps) was below 18 ms, with an additional activation delay of 6 ms introduced by the IDmit module.

These empirical values confirm the suitability of the analytical threshold *t** = 0.43 s, derived from Equations (A1) and (A2).

(A2) $$t^{*}=\frac{1}{\mu -\lambda }ln(\frac{\mu }{\lambda })$$

This expression is useful for real-time estimation of the cutoff threshold.

### Appendix A.2. Pseudocode of Semantic Injection Module

The following pseudocode illustrates the behavior of module 𝒪_4_ in the (H-DIR)^2^ pipeline. It governs the semantic enrichment of RDF graphs based on anomaly scores and entropy deviation as follows:

For each node i:

if ΔH(i) > τ and a_i(t) > θ then

G = AddTriple(G, (Node_i, isLikelyUnderAttack, Attack_Type))

This rule-based injection operator ensures that only statistically significant anomalies are promoted to symbolic assertions. It operationalizes the transition from numerical detection to explainable semantic representation, enabling downstream tasks such as SPARQL reasoning, policy mapping, and ontology-based traceability.

Reproducibility

To ensure full reproducibility of our results, we provide all Jupyter notebooks, preprocessed datasets, and configuration files used in the (H-DIR)^2^ architecture experiments (SYN Flood, DAO-DIO, and NTP Amplification) via a public GitHub repository. All materials are available at the following link: https://github.com/RobUninsubria/HDIR2-paper.git (accessed on 29 June 2025).

**Table A1:** Figures and captions used throughout the manuscript.

Fig.	Label	Caption
1	fig:workflow	(H-DIR)^2^ framework simulation pipeline. Operators O_0_ → O_5_ represent RDF ingestion, Spark SQL streaming, vectorization, ARNN prediction, semantic enrichment, and dynamic update.
2	fig: dual scalability	Dual scalability of the (H-DIR)^2^ architecture. Vertical scalability (Spark, RDD, Minikube) vs. horizontal semantic scalability (SPARQL, RDF, ARNN).
3	fig:dual-level-cycle	Semantic-ARNN coupling. Dynamic loop between semantic rules and ARNN constraints, with ΔH > τ triggering adaptation.
4	fig:etl-bridge	The ψₛₑₘ_→_ₙₑᵤ operator maps semantic triples into the ARNN input space, while the neural outputs are subsequently re-encoded as RDF triples.
5	fig: graph-matrix	Neural attack graph construction. Nodes flagged as critical if ∑_j_ w_ij_ > γ, enabling initiative-taking mitigation and risk propagation.
6	fig:dao-rpl	DAO DIO attack visualization. Entropy drift and semantic feedback highlight abnormal loops in IPv6 RPL topologies.
7	fig: dao-routing	The center panel illustrates the impact of a DAO-DIO manipulation attack, which increases disorder and creates abnormal links.
8	fig: NTP amp	The framework semantically annotates TTL drift, route deviation, and back-end packet surge using RDF/SPARQL and subsequently visualizes them for analysis.
9	fig: NTP amp	Case study
10	fig: scalability-stress	Throughput and Latency vs. Device Count
11	fig: semantic graph	Semantic-to-Adaptive graph interaction. Conceptual diagram showing how RDF/SPARQL assertions affect node-level predictions via ψ_sem→neu, and how ARNN updates feedback into semantic enrichment via ψ_neu→sym.

Each figure is consistently labeled, cross-referenced, and contextually integrated throughout the manuscript.
